# Diagnostic and societal impact of implementing the syncope guidelines of the European Society of Cardiology (SYNERGY study)

**DOI:** 10.1186/s12916-023-03056-6

**Published:** 2023-09-25

**Authors:** M. Ghariq, W. B. van den Hout, O. M. Dekkers, M. Bootsma, B. de Groot, J. G. J. Groothuis, M. P. M. Harms, M. E. W. Hemels, E. C. A. Kaal, E. M. Koomen, F. J. de Lange, S. Y. G. Peeters, I. A. van Rossum, J. H. W. Rutten, E. W. van Zwet, J. G. van Dijk, R. D. Thijs, on behalf of the SYNERGY Consortium, R. M. Tuinema, W. Voet, D. B. Boerman, M. Firouzi, C. Fokke

**Affiliations:** 1 Department of Neurology, Leiden University Medical Centre, PO Box 9600, 2300 RC Leiden, The Netherlands; 2 Department of Biomedical Data Sciences, Leiden University Medical Centre, Leiden, The Netherlands; 3 Department of Clinical Epidemiology, Leiden University Medical Centre, Leiden, The Netherlands; 4 Department of Cardiology, Leiden University Medical Centre, Leiden, The Netherlands; 5 Department of Emergency Medicine, Leiden University Medical Centre, Leiden, The Netherlands; 6 Department of Cardiology, Diakonessenhuis, Utrecht, The Netherlands; 7 Department of Internal Medicine, University Medical Centre Groningen, Groningen, The Netherlands; 8 Department of Cardiology, Rijnstate Hospital, Arnhem, The Netherlands; 9 Department of Cardiology, Radboud University Medical Centre, Nijmegen, The Netherlands; 10 Department of Neurology, Maasstad Hospital, Rotterdam, The Netherlands; 11 Department of Cardiology, Gelre Hospital, Apeldoorn, The Netherlands; 12 Department of Cardiology, Amsterdam University Medical Centre, Amsterdam, The Netherlands; 13 Department of Emergency Medicine, Flevo Hospital, Almere, The Netherlands; 14 Department of Internal Medicine, Radboud University Medical Centre, Nijmegen, The Netherlands; 15 Stichting Epilepsie Instellingen Nederland, Heemstede, The Netherlands

**Keywords:** Transient loss of consciousness, Healthcare efficiency, Syncope, Epilepsy, Admission, Tilt table testing, QALY

## Abstract

**Background:**

Syncope management is fraught with unnecessary tests and frequent failure to establish a diagnosis. We evaluated the potential of implementing the 2018 European Society of Cardiology (ESC) Syncope Guidelines regarding diagnostic yield, accuracy and costs.

**Methods:**

A multicentre pre-post study in five Dutch hospitals comparing two groups of syncope patients visiting the emergency department: one before intervention (usual care; from March 2017 to February 2019) and one afterwards (from October 2017 to September 2019). The intervention consisted of the simultaneous implementation of the ESC Syncope Guidelines with quick referral routes to a syncope unit when indicated. The primary objective was to compare diagnostic accuracy using logistic regression analysis accounting for the study site. Secondary outcome measures included diagnostic yield, syncope-related healthcare and societal costs. One-year follow-up data were used to define a gold standard reference diagnosis by applying ESC criteria or, if not possible, evaluation by an expert committee. We determined the accuracy by comparing the treating physician’s diagnosis with the reference diagnosis.

**Results:**

We included 521 patients (usual care, *n* = 275; syncope guidelines intervention, *n* = 246). The syncope guidelines intervention resulted in a higher diagnostic accuracy in the syncope guidelines group than in the usual care group (86% vs.69%; risk ratio 1.15; 95% CI 1.07 to 1.23) and a higher diagnostic yield (89% vs. 76%, 95% CI of the difference 6 to 19%). Syncope-related healthcare costs did not differ between the groups, yet the syncope guideline implementation resulted in lower total syncope-related societal costs compared to usual care (saving €908 per patient; 95% CI €34 to €1782).

**Conclusions:**

ESC Syncope Guidelines implementation in the emergency department with quick referral routes to a syncope unit improved diagnostic yield and accuracy and lowered societal costs.

**Trial registration:**

Netherlands Trial Register, NTR6268

**Supplementary Information:**

The online version contains supplementary material available at 10.1186/s12916-023-03056-6.

## Background

Syncope is the type of transient loss of consciousness (TLOC) that is due to global cerebral hypoperfusion; it is characterised by a rapid onset, short duration and complete and spontaneous recovery [1]. Syncope is very common, affecting up to 40% of all people at least once in a lifetime. It accounts for ~ 1% of all emergency department (ED) visits and is associated with high healthcare costs [2–5]. The differential diagnosis is broad, and the causes of syncope range from benign to life-threatening conditions [1, 6]. Recurrent syncope has a significant impact on quality of life [7]. Effective diagnosis and treatment of the underlying may reduce this burden [1, 7].

There is huge variation in the management of syncope [8, 9]. The diagnosis is often inaccurate, inefficient, delayed or unknown [10]. Although vasovagal syncope is the most common cause of TLOC, it is not claimed by any specialty nor taught in detail [1, 11, 12]. As a result, specialists often restrict their evaluation to tests for those disorders within their own specialty. Neurologists may, for example, focus on tests for epilepsy, while cardiologists concentrate on tests for arrhythmias and structural heart disease [6, 11].

The 2018 European Society of Cardiology Syncope (ESC) Syncope Guidelines provide guidance for the initial syncope evaluation, risk stratification and follow-up of syncope [1]; the latter includes the availability of a dedicated multidisciplinary syncope unit [13–18]. Implementation of earlier versions of the ESC Syncope Guidelines in several European countries increased diagnostic yield and accuracy [9, 19–28]. Such studies are, as yet, lacking the methodology of comparing two sequential groups and also lacking for the latest most recent 2018 ESC Syncope Guidelines. Another understudied aspect of syncope care includes the added value of quick follow-up at the syncope unit.

The SYNERGY pre-post study (short for ‘SYNcope algorithms in the EmeRGgencY department with structured follow-up’) evaluated the simultaneous implementation of the ESC Syncope Guidelines in the ED together with quick access to a dedicated outpatient syncope unit. We compared diagnostic accuracy as the primary outcome before and after this intervention in a multicentre pre-post study involving five Dutch hospitals. We also investigated diagnostic yield, syncope-related healthcare and societal costs.

## Methods

### Design

We conducted a prospective multicentre pre-post study (Netherlands Trial Register, NTR6268 (old register)/https://onderzoekmetmensen.nl/nl/trial/23988 (new register)) comparing usual care (i.e. period prior to intervention), with the period after the syncope guidelines intervention. Five Dutch hospitals participated, including one university hospital (Leiden University Medical Centre, Leiden) and four regional ones (Diakonessenhuis, Utrecht; Rijnstate Hospital, Arnhem; Gelre Hospital, Apeldoorn; and Maasstad Hospital, Rotterdam).

The syncope guidelines intervention included two components: (1) implementation of the ESC 2018 Syncope Guidelines in the ED for all specialties by educating all involved healthcare providers and (2) establishment of a multidisciplinary syncope unit with quick referral routes for outpatient evaluation if needed [10].

### Population

Patients were enrolled between March 2017 and September 2019 and followed for 1 year. Patients were included 24 h per day and 7 days a week. The study started sequentially in each centre, with intervals of 2 to 4 months between each centre. The usual care and intervention periods lasted 6 months or longer to reach the inclusion target per centre. After completion of the usual care period, we paused the study enrolment for several weeks to educate the ED staff. All patients ≥ 18 years with suspected syncope visiting the ED were eligible for inclusion. We provided an operational definition for ‘suspected syncope’ to standardise clinical recognition in the ED. Suspected syncope was defined as TLOC that was (1) not due to traumatic head injury, (2) with no characteristics specific for seizure (e.g. post-ictal confusion, lateral tongue bite, > 20 jerks) and (3) with no characteristics specific for psychogenic TLOC (e.g. long duration, eyes closed during TLOC) [1, 29]. The exclusion criteria were (1) serious life-threatening conditions (e.g. massive bleeding; pulmonary embolus), (2) inability to give consent, (3) presyncope only and (4) syncope evaluation in the ED in the previous year. We also excluded cases in the usual care group who were referred to a syncope unit outside the study region as this included a component of the intervention. Participants were recruited 24 h per day and 7 days per week. We did not employ paid research staff. The PI of every site reminded all residents and specialists at daily rounds of the study. Residents and specialists recruited study participants. If a patient expressed interest, the patient received an information letter and a notification was sent to the researcher who contacted the subject after at least 24 h to provide additional explanation and to ask the subject to sign the informed consent letter if the subject agreed to participate.

### Syncope guidelines intervention

#### ESC Syncope Guidelines implementation

The ESC Syncope Guidelines were implemented in the ED to structure the evaluation of suspected syncope in a stepwise manner: (1) recognition of TLOC and differentiation between syncope and non-syncopal TLOC, (2) initial syncope evaluation (i.e. structured history taking, physical examination, supine and upright blood pressure measurements and a 12-lead ECG) and (3) risk stratification in those without a certain or highly likely diagnosis (1). Management strategies differed by risk (1). Those with low-risk criteria only were discharged home or referred to the general practitioner; those with any high-risk factor for cardiac syncope were evaluated by a cardiologist in the ED; those with intermediate risk could be referred to the syncope unit or admitted for further evaluation, which choice was left to clinical judgement. Syncope unit referral was also recommended for those with intermediate risk following an uneventful clinical observation period or for those with exclusively low-risk features but recurrent and incapacitating syncopal events.

Prior to the intervention period, no participating hospital had implemented the ESC Syncope Guidelines in the ED, and none of the EDs referred to a multidisciplinary syncope unit. Pre-intervention syncope care differed between sites. The primary evaluation was performed by emergency physicians or cardiologists (Leiden University Medical Centre, Diakonessenhuis Utrecht and Rijnstate Hospital), internists specialised in acute medicine or cardiologists (Maasstad Hospital), or internists, cardiologists or neurologists (Gelre Hospital). In all centres, patients were primarily seen by a resident supervised by a specialist.

We organised teaching sessions of 2 h explaining the ESC guidelines as part of the implementation. These sessions were aimed at all residents and specialists involved in syncope care in the ED and were repeated at least three times in each hospital to ensure that all relevant personnel could take part. Flash cards with ESC Syncope Guidelines flowcharts were distributed among the ED staff. Sessions were presented by a syncope specialist (SYGP or other medical specialists with expertise in syncope and knowledge of ESC syncope guidelines). New residents starting work at the ED during the study were educated individually. Nurses, technicians and medical specialists working at the syncope unit attended a 1-day course at the Leiden University Medical Centre. All centres adhered to the EFAS/European Academy of Neurology protocol for tilt testing [30].

#### Quick referral routes to a syncope unit

All participating hospitals established a multidisciplinary syncope unit meeting the ESC/European Heart Rhythm Association (EHRA) standards prior to the intervention phase [10]. All units had access to all required diagnostic facilities for the evaluation of TLOC, including a continuous blood pressure monitor for tilt testing and the active standing test [10, 14, 30]. Operating procedures of cardiovascular autonomic tests accorded with European Federation of Autonomic Societies (EFAS) standards [30, 31]. The newly established syncope units were headed by one cardiologist and one neurologist with expertise in syncope care. All syncope units organised regular multidisciplinary meetings and prioritised ED referrals by offering visits within 2 weeks. The neurology departments of two hospitals (Rijnstate Hospital and Leiden University Medical Centre) already had tilt facilities before the start of the study, but without preferred ED referral and no formalised multidisciplinary approach, both of which were addressed prior to the intervention phase.

### Reference standard

**Fig. 1:**
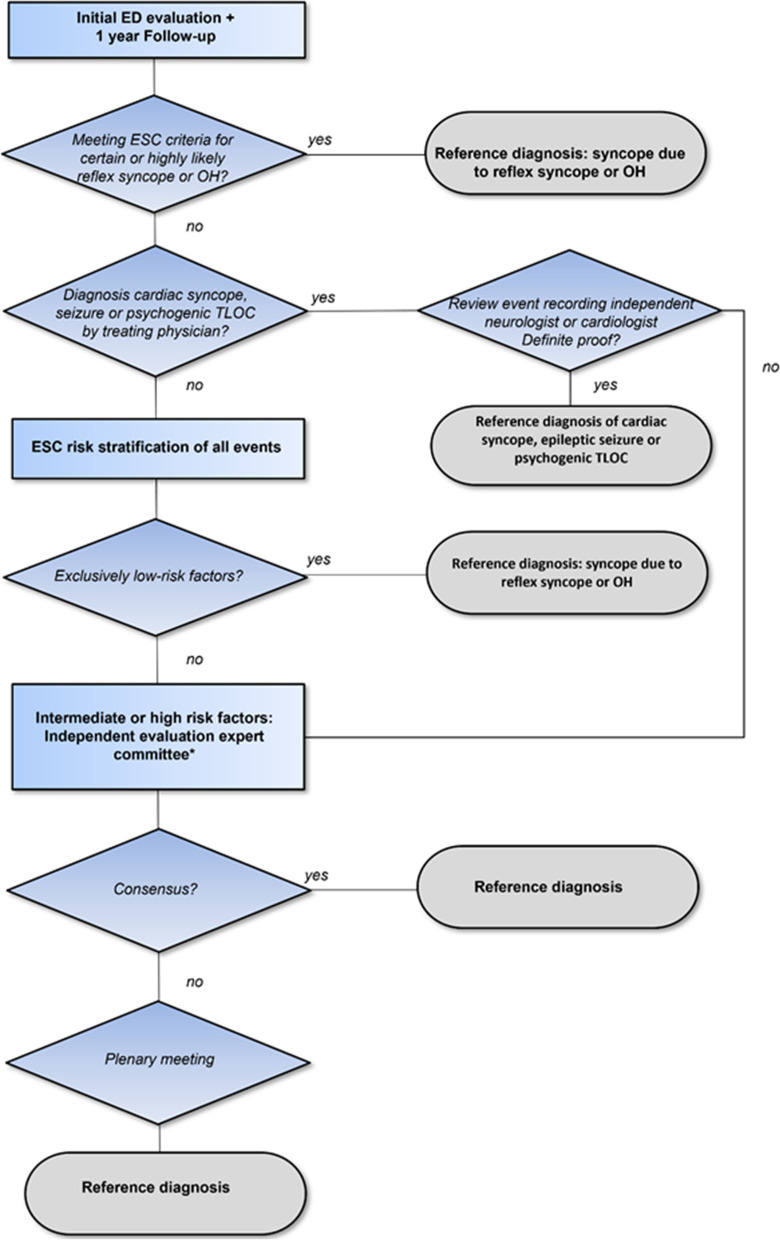
Determination of reference diagnosis after 1 year of follow-up. All case files were reviewed to evaluate whether the initial event and all possible recurring events met the ESC criteria for a certain/highly likely diagnosis for reflex syncope or syncope due to orthostatic hypotension. If the treating physician issued a diagnosis of psychogenic TLOC, epilepsy or cardiac syncope, the case files were reviewed by a member of the expert committee to assess whether definite proof was obtained or not (cardiac syncope: heart rate recording; psychogenic TLOC/epilepsy: video-EEG). If the proof was not definite, the case was reviewed by the multidisciplinary expert committee. This committee also evaluated all cases that were stratified as intermediate risk or high risk. *Expert committee consisted of one cardiologist, one neurologist and one internist

We used 1-year follow-up data to determine the gold standard final diagnosis called the ‘reference diagnosis’ (Fig. 1) [32]. We first gathered all clinical information pertaining to the 1 year of follow-up. All participants received a questionnaire at baseline and at 3, 6 and 12 months of follow-up including questions on TLOC recurrence and doctor visits. If the patient reported TLOC recurrence evaluated in a non-participating hospital or by a general practitioner, we retrieved the clinical notes. A research nurse presented all medical information in a case record form stripped of identifying data to ensure blinding.

Next, cases were evaluated by applying the ESC criteria. All cases were evaluated by the research physician (MG) and, in case of doubt, by RDT. If the initial and any recurring events met the ESC criteria for a certain or highly likely diagnosis for reflex syncope or syncope due to orthostatic hypotension, those were used as reference diagnosis. When the treating physician issued a diagnosis of psychogenic TLOC, epilepsy or cardiac syncope, we reviewed the notes to assess whether definite proof was obtained: cases with psychogenic TLOC and epilepsy were evaluated by a neurologist considering video EEG as definite proof and cardiac syncope by a cardiologist considering ECG or echocardiographic findings as definite proof.

The next step involved the application of the ESC syncope risk stratification to cases without a definite diagnosis (Fig. 1). If patients had low-risk criteria only, without any recurrence during follow-up suggesting another diagnosis, the event was classified as ‘syncope due to reflex syncope or orthostatic hypotension’. Reflex syncope and orthostatic hypotension (OH) were bundled as one major ‘blood pressure related’ category, in view of common features in pathophysiology and risks.

Finally, if the data did not allow definite proof, the case was reviewed by the expert committee. Committees differed per occasion but always comprised one cardiologist (MB, MEWH or FdL), one neurologist (RDT, JGvD, IvR or EK) and one internist (JR or MPMH). All members had extensive experience in syncope care. All cases were first assessed independently by members of the expert committee, using predefined categories (Additional file 1): when they all agreed, their opinion was used as the final diagnosis; if not, consensus was reached in face-to-face or virtual meetings. We used Krippendorff’s alpha test to estimate the inter-rater agreement for the three raters in each consensus panel [33].

#### Classification of the treating physician’s diagnosis

The treating physician’s diagnosis was classified according to the predefined criteria (Table 1). We also considered the timing of the diagnosis as the treating physician as well as the diagnosis may vary over time. When a patient had multiple hospital consultations, we evaluated the notes of the last treating physician. If the diagnosis was concordant with the management plan, we recorded this diagnosis as the treating physician’s diagnosis. If the diagnosis from ED was not concordant with the management plan, we classified the case as ‘unexplained syncope’. For example, a conclusion of ‘possible arrhythmia’ without documentation of arrhythmia and no appropriate management (e.g. follow-up or treatment) was labelled as ‘unexplained syncope’. If, however, a diagnosis of ‘cardiac syncope’ was accompanied by management aimed at cardiac causes (e.g. implantable loop recorder or pacemaker implantation), we maintained this diagnosis. All cases were evaluated by the research physician (MG) and discussed, in case of doubt, with RDT. Survival status was determined using the electronic patient records. Reported deaths were evaluated by MG and RDT and classified as syncope-related deaths or non-syncope-related deaths.

**Table 1:** Criteria to determine the treating physician’s diagnosis

**Treating physician’s conclusion**	**Criteria to evaluate concordance of the management plan with the suggested condition**			
	**Admission**	**Follow-up**	**Investigations following ED visit**	**Treatment**
Reflex syncope (including vasovagal syncope, carotid sinus syndrome and situational syncope)	No admission except for underlying cause (e.g. gastroenteritis) or trauma due to syncope	No referral to outpatient clinic, except for syncope unit	No investigations except for tilt test (*optional*)	Optional: Education in counter pressure manoeuvres Treatment with drugs that may prevent reflex syncope (e.g. midodrine)
Orthostatic hypotension (including initial, classic and delayed orthostatic hypotension)	No admission except for underlying cause (e.g. dehydration, bleeding)	Optional: Referral to GP or outpatient clinic for analysis of underlying cause	Optional: Tilt table testing, active standing test, autonomic function test, work-up to identify underlying neurological cause (e.g. polyneuropathy)	Optional: Treatment of underlying cause (e.g. rehydration) Deprescribing of blood pressure-lowering drugs Prescription of blood pressure-increasing drugs Education in counter-pressure manoeuvres
Cardiac syncope Cardiopulmonary and great vessels	Admission to cardiology department* except when treatment did not necessitate admission Admission if needed	Follow-up cardiology department to confirm diagnosis or evaluate treatment Follow-up pulmonologist, vascular surgeon	Optional: Monitoring heart rhythm (in-hospital, Holter ECG) Echocardiography Exercise testing Implantation of cardiac monitoring devices Imaging aorta/pulmonary veins	Optional: Implantation of pacemaker/defibrillator Surgical intervention for structural causes Prescription of anti-arrhythmical drugs Optional: Anti-thrombotic therapy Surgical intervention
Epileptic seizure	Optional: Admission to neurology department	Referral to neurology outpatient clinic except for provoked seizures	Optional: MRI, CT brain or EEG	Optional: Prescription of anti-seizure medication
Psychogenic TLOC	No admission except for injuries due to TLOC necessitating admission	Variable (no follow-up, referral to GP, consultation psychiatrist or psychologist)	None	Education or treatment plan as defined by psychiatrist, psychologist or GP

The classification of the treating physician’s diagnosis was based on the final conclusion in the medical notes and the chosen diagnostic or therapeutic pathway. Concordance between the presumed diagnosis and the management plan was evaluated using predefined criteria for all causes of TLOC. If the treating physician’s conclusion was concordant with the management plan, then this diagnosis was labelled as the treating physician’s diagnosis. If the diagnosis was not concordant with the management plan, we classified the treating physician’s diagnosis as unexplained syncope or TLOC

*Abbreviations*: *GP* General practitioner, *OH* Orthostatic hypotension, *CT* Computed tomography, *MRI* Magnetic resonance imaging, *EEG* Electroencephalogram, *TLOC* Transient loss of consciousness

^*^Or another department with facilities to continuously monitor heart rate

### Diagnostic accuracy

Our primary outcome measure was diagnostic accuracy. Diagnostic accuracy was defined as the proportion of cases with a correct diagnosis of TLOC within 1 year of follow-up. We determined the accuracy of the diagnosis by evaluating the concordance between the treating physician’s diagnosis (as described above) and the reference diagnosis. We only labelled diagnoses as discordant if the inconsistency occurred at the level of major TLOC categories (i.e. blood pressure-related causes of syncope, including reflex syncope and all forms of OH; cardiac syncope; epilepsy; psychogenic TLOC or TLOC of unknown cause; Additional file 1). For example, if the reference diagnosis was reflex syncope and the treating physician’s diagnosis was psychogenic TLOC, we labelled the diagnoses as discordant (i.e. inaccurate). Inaccuracies within major TLOC categories, such as orthostatic hypotension and reflex syncope (Additional file 1), were treated as accurate in our primary analysis of diagnostic accuracy. We also examined the accuracy of the subclassification of blood pressure-related syncope by the treating physician as a secondary outcome measure.

### Syncope-related healthcare and societal costs

Secondary outcome measures included diagnostic yield, syncope-related admissions, tests, healthcare costs, societal costs and quality-adjusted life years (QALYs) in the year following the initial ED visit. We determined the diagnostic yield by calculating the proportion of patients who received a diagnosis of TLOC within 1 year of follow-up in each cohort (usual care and syncope guidelines intervention). To calculate the syncope-related healthcare costs and societal costs, we derived healthcare utilisation data from the patient records of the hospital where the initial ED visit took place. We also calculated healthcare costs in other hospitals, GP care and days lost from paid work to syncope with questionnaires, filled out by the patients at 3, 6 and 12 months. We selected all healthcare costs (e.g. diagnostic tests, therapies, hospital visits and admissions) directly related to the management of syncope (e.g. ECG) as well as indirect syncope-related healthcare costs (e.g. X-ray for trauma due to syncope). The latter category also included travel costs. Syncope-related societal costs were calculated by adding the costs of absenteeism to the healthcare expenditures. All costs were valued according to Dutch guidelines for economic healthcare evaluations, using Dutch reference prices if available or prices at the LUMC [34]. We reported all costs in Euros, at the price level of 2021. We calculated utilities from the EQ-5D-5L using the Dutch tariff, representing the value of quality of life on a scale from 0 (as bad as death) to 1 (perfect health) [35]. QALYs were calculated for each patient as the area under the utility curve.

### Sample size

The five EDs together saw ~ 166,000 patients per year (Leiden University Medical Centre: 30,000; Rijnstate Hospital: 38,000; Maasstad Hospital: 45,000; Gelre Hospital: 27,000; Diakonessenhuis: 26,000). A 1% syncope prevalence in the ED would result in ~ 1660 eligible patients per year [2–5, 18]. We anticipated a diagnostic yield of 64% for usual care [32] and 80% for the intervention [10]. Assuming similar accuracy for both groups and an intervention effect of 16% (SD 3), we expected to detect significant differences across the five participating EDs with a power of 0.98 and on average across the general population with a power of 0.8 by including 275 patients per arm (two-sided alpha of 0.05).

### Statistical analysis

We used IBM SPSS Statistics 23 for diagnostic analyses. Continuous data were expressed as mean (standard deviation) or median (interquartile range) when appropriate and categorical data as counts (proportion). We analysed the between-group differences in continuous data using Student’s *t*-test or non-parametric tests where appropriate, and we applied a logistic regression model to estimate the (log) risk ratio to compare diagnostic accuracy between both groups (syncope guidelines intervention and usual care) with fixed effects per centre. We used Stata/IC 14.2 for Windows to compare costs, using regression analysis with multiple imputation to account for missing questionnaire data (100 imputed data sets, with random effects per centre). We expressed the effect sizes for the main outcome measures as 95% confidence intervals. Statistical significance was set at *p* < 0.05.

## Results

### Study population

A total of 548 patients gave informed consent between 1 March 2017 and 1 September 2019 (Additional file 2). Fourteen patients in the usual care group were excluded because they visited a syncope unit. Thirteen patients were excluded because in retrospect they had presyncope, leaving 275 patients in the usual care group and 246 patients in the syncope guidelines group. Most patients were 50 years or older (mean age 63 ± 17 in the usual care group and 64 ± 16 in the syncope algorithm group) and had a diagnosis of reflex syncope or OH according to the reference standard. The distribution of age, gender and reference diagnosis was comparable between the groups (Table 2).

**Table 2:** Patient characteristics, clinical features and diagnostic tests in the usual care and the syncope guidelines groups

	**Usual care, *n* = 275**	**Syncope guidelines, *n* = 246**	***p*-value**	**Missing data**
**Age, years (mean, SD)**	63 ± 17	64 ± 16	0.44	None
**18–35 years**	24 (8.7%)	15 (6.1%)		
**36–50 years**	35 (13%)	32 (13%)		
**51–74 years**	139 (51%)	129 (52%)		
**> 75 years**	77 (28%)	70 (29%)		
**Sex**			0.68	None
**Male**	157 (57%)	136 (55%)		
**Female**	118 (43%)	110 (45%)		
**Centre**			0.23	None
**Leiden**	40 (15%)	50 (20%)		
**Utrecht**	56 (20%)	55 (22%)		
**Arnhem**	48 (18%)	39 (16%)		
**Rotterdam**	61 (22%)	56 (23%)		
**Apeldoorn**	70 (26%)	46 (19%)		
**Diagnosis according to the reference standard**			0.19	None
**Syncope due to reflex syncope or OH**	198 (72%)	193 (79%)		
**Cardiac syncope**	26 (9.5%)	25 (10%)		
**Epileptic seizure**	9 (3.3%)	5 (2.0%)		
**Psychogenic TLOC**	5 (1.8)	1 (0.4%)		
**Unknown aetiology**	37 (14%)	22 (8.9%)		
**Time of ED visit**			0.36	None
**12 am–6 am**	21 (7.6%)	26 (11%)		
**6 am–12 pm**	88 (32%)	65 (26%)		
**12 pm–6 pm**	111 (40%)	99 (40%)		
**6 pm–12 am**	54 (20%)	56 (23%)		
**Referral pathway**			0.90	None
**Self-referral**	15 (5.5%)	10 (4.1%)		
**GP referral**	53 (19%)	50 (20%)		
**Ambulance**	183 (67%)	164 (67%)		
**Not documented**	24 (8.7%)	22 (8.9%)		
**Manchester Scale Triage code**			0.44	None
**Red**	–	1 (0.4%)		
**Orange**	20 (7.3%)	16 (6.5%)		
**Yellow**	165 (60%)	152(62%)		
**Green**	47 (17%)	31(13%)		
**Unknown**	43 (16%)	46 (19%)		
**Family history of sudden cardiac death (< 60 years)**			0.25	None
**Presence or absence mentioned**	38 (14%)	43 (17%)		
**Presence or absence not mentioned**	237 (86%)	203 (83%)		
**Posture prior to TLOC**			0.88	None
**Mentioned**	238 (87%)	214 (87%)		
**Not mentioned**	37 (14%)	32 (13%)		
**Confusion afterwards**			0.16	None
**Presence or absence mentioned**	200 (73%)	192 (78%)		
**Presence or absence not mentioned**	75 (27%)	54 (22%)		
**Tongue bite**			0.45	None
**Presence or absence mentioned**	200 (73%)	186 (76%)		
**Presence or absence not mentioned**	75 (27%)	60 (24%)		
**Prodromes**			1.00	None
**Presence or absence mentioned**	275 (100%)	246 (100%)		
**Presence or absence not mentioned**	–	–		
**Circumstances related to TLOC**			0.41	None
**Mentioned**	257 (94%)	234 (95%)		
**Not mentioned**	18 (6.5%)	12 (4.9%)		
**ECG in the ED**			0.20	None
**Recorded**	254 (92%)	234 (95%)		
**Not recorded**	21 (7.6%)	12 (4.9%)		
**Holter ECG**				
**Recorded**	28 (10%)	23 (9.3%)	0.75	
**Not recorded**	247 (90%)	223 (91%)		
**OH screening (standing test) in the ED**			**< 0.01**	None
**Performed**	45 (16%)	64 (26%)		
**Not performed**	230 (84%)	182 (74%)		
**No. of consultations in the ED**			0.30	None
**One**	131 (48%)	121 (50%)		
**Two**	101 (37%)	74 (30%)		
**Three**	34 (12%)	40 (16%)		
**Four**	9 (3.3%)	11 (4.5%)		
**Hospital admission following ED visit**	146 (53%)	106 (43%)	**0.02**	None
**With telemetry**	110 (40%)	87 (35%)		
**Without telemetry**	36 (13%)	19 (7.7%)		
**Hospital admissions following ED visit among those with exclusively low-risk criteria**				
**Yes**	84 (43.1%)	66 (34%)	**0.07**	None
**No**	111 (57%)	127 (66%)		
**Syncope unit referral**	–	37 (15%)	Not performed	None
**Tilt testing**	5 (1.8%)	17 (6.9%)	**< 0.01**	None
**Implantation loop recorder**	10 (3.6%)	13 (5.3%)	0.36	None
**QALY for the study period (EQ5D)**	0.81	0.79	0.50	UC 104 (37%)
				SG 113 (46%)

*Abbreviations*: *GP* general practitioner, *OH* orthostatic hypotension, *TLOC* transient loss of consciousness, *ED* emergency department, *QALY* quality-adjusted life years, *EQ5D* EuroQol-5 Dimension, *UC* usual care, *SG* syncope guidelines

Five non-syncope-related deaths were recorded during follow-up: three in the usual care group (due to choking, gastrointestinal bleeding and urosepsis later during the follow-up) and two in the syncope guidelines group (postoperative brain infarction and postoperative pulmonary embolus).

### Diagnostic performance

**Fig. 2:**
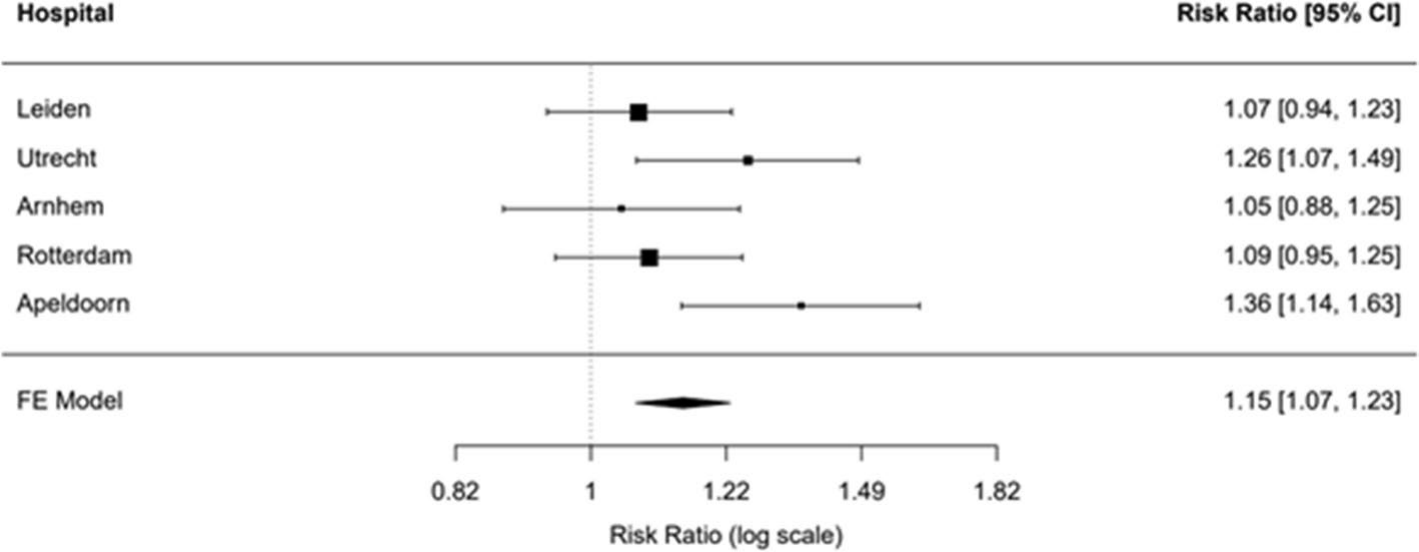
Fixed effects model of diagnostic accuracy. The treating physician’s diagnosis matched the reference diagnosis more frequently in the syncope guidelines intervention group than in the usual care group. UC, usual care; SG, syncope guidelines

**Fig. 3:**
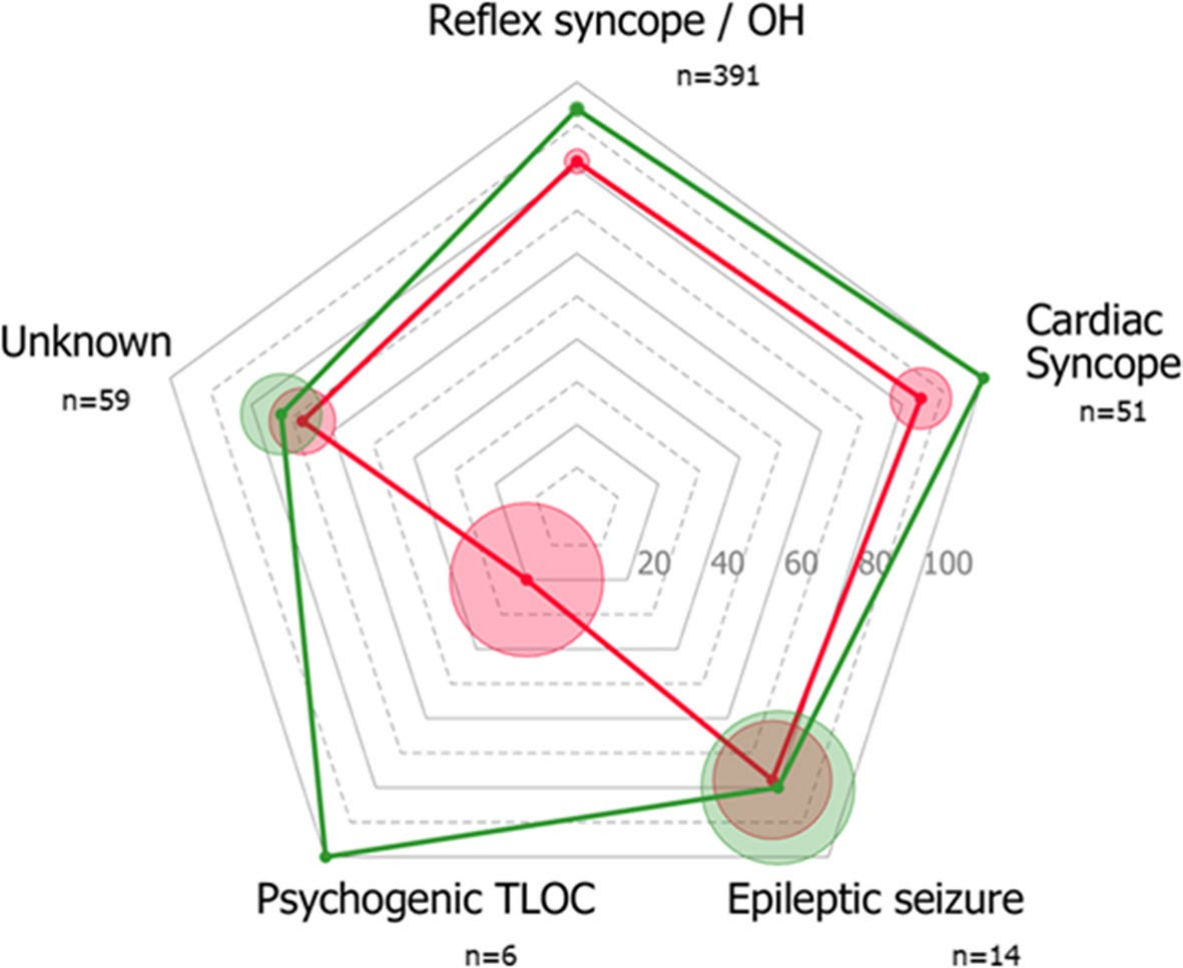
Radar chart of the diagnostic accuracy of the treating physician’s diagnosis for each major cause of TLOC. Diagnostic accuracy was expressed as the proportion of cases with a correct diagnosis according to the reference standard. Data are presented per diagnostic category for the usual care group (red line) and the syncope guidelines (green line). For each category, the total number of cases in both groups is given. The radius of the circles reflects the standard error of the proportion. OH, orthostatic hypotension; TLOC, transient loss of consciousness

**Fig. 4:**
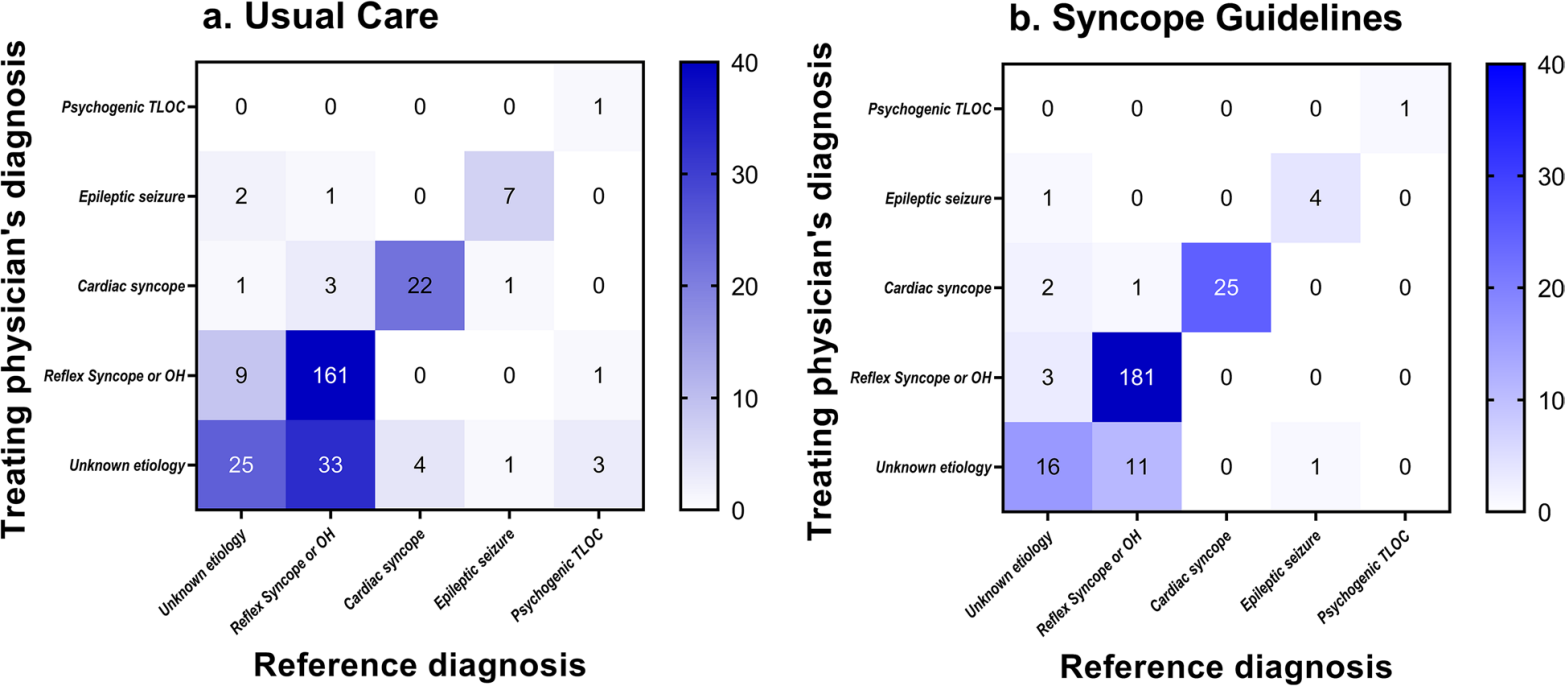
Heat plot expressing the concordance between the diagnosis of the treating physician with the reference diagnosis for two cohorts presenting with syncope in the emergency department: usual care group (left panel) and syncope guidelines intervention group (right panel). OH, orthostatic hypotension; TLOC, transient loss of consciousness

The diagnosis of the treating physician matched the reference diagnosis more frequently in the syncope guidelines group (*n* = 211, 86%) than in the usual care group (*n* = 191, 69%) (risk ratio 1.15 (95% CI 1.07 to 1.23; Fig. 2). The concordance between the treating physician’s diagnosis and the reference diagnosis improved across all major diagnostic categories with most marked improvements for those with blood pressure-related syncope (Figs. 3 and 4, Additional file 3). Diagnostic yield was higher (thus the proportion of unexplained syncope lower) in the syncope guidelines group compared to usual care (89% vs. 76%; 95% CI of the difference 6 to 19%).

### Ancillary testing

The proportion of patients admitted directly from the ED was higher in the usual care group than in the syncope guidelines intervention group (53 vs. 43%; 95% CI of the difference: 2 to 19%). The reduction was most marked among those with exclusively low-risk criteria (usual care 43%; syncope guidelines 34%). Screening for OH in the ED was more frequently performed in the syncope guidelines group compared to usual care (26% vs. 16%, 95% CI of the difference: 3 to 17%). Fifteen per cent of all cases in the syncope guidelines group were referred to the syncope unit. We observed a modest increase in tilt table testing in the syncope guidelines group compared to usual care (7% vs. 2%; 95% CI of the difference: 2 to 9%). The proportion of cases referred for cardiac evaluations (Echocardiogram, Holter-EKG or implantable loop recorders) did not differ between the groups.

A total of 271 of the 521 cases met the ESC criteria for a certain or highly likely diagnosis for reflex syncope or syncope due to orthostatic hypotension. The definite diagnosis of the treating physician of cardiac syncope met our predefined criteria in all 13 cases. The remaining 247 cases without definite diagnosis were evaluated by our blinded consensus panel. The median interrater agreement of all five rounds of the consensus panel yielded a Krippendorf’s alfa statistic of 0.35 (range 0.27–0.41). In 102 out of 247 cases, all three independent raters unanimously agreed. In 135 cases, the final diagnosis was issued following a consensus meeting.

### Healthcare and societal costs

The questionnaire data were complete in 246 cases; in 255 cases, we lacked one or more follow-up questionnaires (108 one, 44 two, 108 three). Syncope-related healthcare costs did not significantly differ between the usual care group (€2393 ± 3844) and syncope guidelines group (€2035 ± 3353, *p* = 0.3) (Table 3), yet the total syncope-related societal costs were lower in the syncope guidelines group compared to usual care (saving of €908 per patient; 95% CI of the difference: €34 to €1782). The QALYs in the year following the initial ED visit were comparable between the groups.

**Table 3:** Syncope-related healthcare costs per group

	Usual care (*n* = 275)		Syncope guidelines (*n* = 246)		Difference	
	Number per patient	Costs per patient	Number per patient	Costs per patient	Costs per patient	*p*-value
Hospital admissions (days)						
Following the initial ED visit*	1.36	727	1.11	591	− 136	0.23
Later	0.91	485	0.74	397	− 88	0.47
Later ED visits	0.16	45	0.15	44	− 1	0.96
Hospital diagnostic procedures	3.35	583	3.08	536	− 47	0.70
Outpatient hospital visits	0.88	94	0.72	78	− 16	0.23
Hospital treatments	0.25	409	0.18	323	− 86	0.64
GP visits	0.71	29	0.96	39	10	0.13
GP diagnostic procedures	0.38	21	0.46	27	6	0.56
**Total healthcare costs (SD)**		**2393 (3844)**		**2035 (3353)**	**− 358**	**0.27**
Absenteeism (days)	5.39	1152	2.62	602	− 550	0.06
**Total societal costs (SD)**		**3545 (5170)**		**2637 (4109)**	**− 908**	**0.04**

Average syncope-related healthcare use, absenteeism and costs (in €) per patient, by study group (usual care, *n* = 275; syncope guidelines, *n* = 246)

## Discussion

### Main findings

Implementation of the ESC 2018 Syncope Guidelines in the ED with appropriate referral routes to a multidisciplinary syncope unit was associated with a higher diagnostic yield and accuracy and lower syncope-related societal costs.

### Strengths and limitations

A major strength of our work includes the combined diagnostic and economic evaluation of the ESC 2018 Syncope Guidelines with a 1-year follow-up in a real-world setting. Our intervention lacked supervised management to control the uptake of the ESC guidelines. As a result, we saw indications of the intervention in some but not all cases. For example, standing to upright blood pressure (BP) measurements in the syncope guidelines group were performed in only about a quarter of all cases, even though the number was significantly higher than in the usual care group. Similarly, the rate of hospital admission declined significantly in the guideline group, but the proportion of admissions remained higher than recommended. Previous studies using obligatory adherence to the guidelines demonstrated a higher increase of diagnostic yield and lower admission rates compared to our work [20, 25] underscoring that the added value of the ESC Syncope Guidelines implementation may be even higher. While the lack of supervised management may be considered a limitation, it reflects a real-world situation and strengthens the generalisability of our findings.

The intervention phase started well after the usual care phase, allowing possible bias due to changing circumstances. The alternative would require parallel pathways, but the two management strategies would then probably not remain separate. We compared diagnoses according to the reference standard in both cohorts and did not identify marked contrasts. Notably, the proportion of cases with cardiac syncope did not differ between both cohorts. We therefore assume that the sample bias was minimal.

We did not ask treating physicians to assign the likelihood of the diagnosis, as we expected that this would introduce an element of the intervention in the usual care period. Prospective evaluation of diagnostic likelihood may also introduce marked variation between physicians, particularly in the context of events with potential serious outcomes where even low risks may prompt ancillary testing to avoid adverse outcomes. Instead, we established criteria to evaluate the management plan and only considered syncope as explained if the final conclusion of the treating physician matched the treatment plan. Another limitation is the lack of a control group to control for secular trend bias [36]. We also considered the subjectivity of the diagnosis as a potential but inevitable limitation. This subjectivity is reflected by the relatively low interrater agreement. We therefore only accepted the expert opinion as a final diagnosis if all three independent raters unanimously agreed. In all other situations, we established multidisciplinary expert groups to obtain consensus. We only included cases with suspected syncope. Although near-syncope is known to confer similar risks for serious clinical events as syncope, we excluded cases with isolated presyncope as we anticipated difficulties in reliably identifying these cases in view of the nebulous nature of the presentation [37].

Healthcare use was assessed using a combination of electronic patient records and patient questionnaires, but we may still have missed some of the expenses in this diverse patient population. Our study is limited to the Dutch healthcare system. Although healthcare savings may differ between countries, the potential of ESC guideline-directed syncope care to save costs has been demonstrated in various healthcare systems [16, 20, 38]. A strength and novelty of our trial is that we assessed the impact of syncope on the absence from work, which emerged as an important driver in the lower syncope-related societal costs in the syncope guidelines group. Another strength is the long-term follow-up incorporating the post-ED stratification workup, allowing us to evaluate the precision of the suggested diagnosis. Our intervention included two components that were implemented simultaneously, so we could not determine the impact of the syncope guidelines implementation and syncope unit establishment separately. We saw, however, signs of both interventions as evaluations that are strongly recommended by the syncope guidelines (e.g. OH measurements) significantly increased in the implementation phase as did the number of syncope unit referrals. Another sign of our intervention concerned the improved diagnostic precision. Our data indicate that the proportion of cases in need for syncope unit referral was ~ 15% [31] confirming the notion that the initial evaluation is sufficient in the majority of cases [1–6, 32, 39]. We need, however, to keep in mind that our trial presents the results immediately following a service delivery intervention. We expect that the referral rate and hereby the added value of the syncope unit on the emergency evaluation of syncope may increase over time.

### Syncope implementation studies

Our findings go beyond previous implementation studies as no studies investigated the simultaneous implementation of the syncope guidelines and quick referrals to a syncope unit [6, 22, 40]. A multicentre study in Italy showed that a structured diagnostic algorithm at the ED based on the syncope guidelines improved the diagnostic yield significantly [19], but without a control group. Another Italian study showed that a standardised care pathway led to a higher diagnostic yield than usual care [20]. Although the study included a control group, the intervention only comprehended the ED evaluation. A recent Dutch study showed that evaluation in the ED by a research physician with strict adherence to the ESC guidelines improved the proportion of correct diagnoses from 65 to 80% [25]. An educational programme promoting the ESC guidelines had only a limited impact on syncope care and did not improve cost-effectiveness, thus underscoring the complexity of guideline implementation in the ED [41]. While education also constituted a key element of our intervention, we additionally offered quick referral routes for patients with intermediate risk. This might explain why our study yielded better results than the former educational intervention study. In a single-centre study in Ireland, ESC Syncope Guidelines implementation was carried out by introducing a local Integrated Care Plan [23]. Local physicians were instructed several times prior to implementation and during 4 weeks afterwards. This resulted in increased appropriate referral rates to the syncope unit. The FAST II study demonstrated the added value of a tertiary syncope unit by demonstrating a high diagnostic yield, accuracy and safety [42, 43]. Notably, those who were diagnosed with reflex syncope underwent a median of nine tests prior to referral. The study also suggested a knowledge gap as often no ancillary testing was needed to yield a highly reliable diagnosis despite multiple consultations by various specialties. Initial orthostatic hypotension was identified as a frequent blind spot which is in line with the improved diagnostic yield for BP-related causes of syncope we found following ESC Syncope Guidelines implementation [42–44]. Similarly, a dedicated outpatient evaluation including tilt testing in cases with unexplained syncope presenting to the ED in Malmö considerably improved diagnostic efficacy and accuracy [45]. As in our study, the majority of cases in this cohort were found to have BP-related causes of syncope including vasovagal syncope, carotid sinus hypersensitivity and orthostatic hypotension. We studied the BP-related causes as a composite outcome as we anticipated difficulties in classification due to incomplete evaluation (e.g. no standing test) or incomplete records (e.g. no mention of the presence or absence of autonomic activation). Although our primary analysis focussed on the accuracy of identifying the major TLOC groups, the classification of BP-related syncope matched the reference diagnosis more frequently in the syncope guidelines group compared to usual care (Additional file 1). In line with previous studies, we found that hospitalisation was the primary driver of syncope-related healthcare costs [2–5, 39]. Annual admission costs exceeded $2.4 billion in the USA, as high as those for HIV and asthma [46, 47]. A recent survey reported higher admission rates among patients with lower-risk syncope in regions with higher malpractice claims, suggesting defensive medicine [4]. While the admission rate significantly declined following our intervention, the overall syncope-related health costs between the groups did not reach significance, probably due to a slight increase in outpatient evaluations in the syncope guidelines group [47]. As our study was powered for diagnostic outcomes, the lack of statistical significance of differences between types of healthcare costs and absenteeism does not allow strong conclusions. However, these differences were mostly in favour of our intervention, and the combined syncope-related societal costs were significantly lower following our intervention.

Previous work suggested that guideline adherence is promoted by combining knowledge transmission, reflective practice and a supportive environment [39, 48]. We, as did others, [49, 50] found implementation of the ESC guidelines to be complex, requiring tailored strategies to overcome potential barriers. While most professionals in our study welcomed the structured workflow in the ED and the multidisciplinary syncope unit as a useful solution to a perceived need in clinical practice, remaining barriers occurred on the level of the individual healthcare professional (e.g. inexperienced residents having to work with the guideline in the ED) and the organisational context (e.g. specialists not relinquishing preceding procedures) [51]. Time pressure emerged in our study as one of the biggest obstacles at the organisational level, while orthostatic BP measurements were often not performed because their yield was perceived as low [51]. The low rate of orthostatic BP measurements in the usual care group (16%) is in line with a previous Dutch survey (16%) [52]. In the latter survey, orthostatic BP measurements were only performed in 48% of those who received a diagnosis of OH, thus questioning the validity of this diagnosis [52]. A US survey found that implementation of new syncope guidelines was seen as a burden, potentially decreasing compliance [49]. Change may be more likely if implementation strategies are specifically chosen to address frequently encountered barriers in physicians’ existing knowledge, attitudes and behaviour. Reliable risk prediction tools are also much needed as they could help to support clinical judgement. Many syncope risk stratification scores have been proposed, yet with conflicting results [6]. A recent multicentre trial reported that early standardised clinical judgement for cardiac syncope in the ED outperformed the frequently used Evaluation of Guidelines in Syncope Study (EGSYS) syncope prediction score [53]. History taking is still the cornerstone of the initial syncope evaluation [6, 31, 54].

## Conclusions

Implementing the ESC 2018 Syncope Guidelines in the ED including quick referral pathways to syncope units led to an increase in diagnostic accuracy, a reduction in the proportion of unexplained syncope and a decrease in societal costs, i.e. better and cheaper care.

### Supplementary Information

**Additional file 1.** List of expert committee diagnoses and corresponding major diagnostic categories. *An ictal asystole due to a focal seizure was classified as “syncope due to arrythmia” as the asystole was the primary cause of TLOC [55]. Abbreviations: TLOC= transient loss of consciousness; OH=orthostatic hypotension.

**Additional file 2.** Study timelines. Start and end dates of the pre-intervention period (Usual Care; yellow) and the ESC Syncope Guidelines intervention (green). The figure depicts the timelines of all five study sites. After completion of the Usual Care period we paused study enrolment to educate the ED staff (yellow-green gradient).

**Additional file 3.** Heat plot expressing the concordance between the classification of blood pressure related syncope by the treating physician and the reference standard for two cohorts presenting with syncope in the emergency department: Usual Care group (left panel) and Syncope Guidelines intervention group (right panel). We selected all cases were the treating physician’s diagnosis of “blood pressure related syncope” matched the reference standard (Usual Care *n*=161; Syncope intervention *n*=181) and present the subclassifications of this category: reflex syncope, orthostatic hypotension or unclassifiable (eg, too little information to reliably differentiate or in case two conditions coincided; for instance vasovagal syncope and initial orthostatic hypotension). Abbreviations: OH = orthostatic hypotension; TLOC= transient loss of consciousness.

## Data Availability

Access to the complete database, containing privacy-sensitive information, requires prior approval from the Medical Research Ethics Committee of the Leiden University Medical Centre and the members of the SYNERGY Consortium.

## Abbreviations

BP: Blood pressure
CT: Computed tomography
ED: Emergency department
EEG: Electroencephalogram
EFAS: European Federation of Autonomic Societies
EGSYS: Evaluation of Guidelines in Syncope Study
EHRA: European Heart Rhythm Association
EQ5D: EuroQol-5 Dimension
ESC: European Society of Cardiology
GP: General practitioner
MRI: Magnetic resonance imaging
OH: Orthostatic hypotension
QALY: Quality-adjusted life years
SYNERGY: SYNcope algorithms in the EmeRGgencY department with structured follow-up
TLOC: Transient loss of consciousness
