# Supplementary material for: Diagnostic and societal impact of implementing the syncope guidelines of the European Society of Cardiology (SYNERGY study)
Source: BMC Med. 2023 Sep 25;21:365. doi: 10.1186/s12916-023-03056-6 (PMC10518933; doi:10.1186/s12916-023-03056-6)
Supplement: Supplementary file 1 — Additional file 1. List of expert committee diagnoses and corresponding major diagnostic categories. *An ictal asystole due to a focal seizure was classified as “syncope due to arrythmia” as the asystole was the primary cause of TLOC [55]. Abbreviations: TLOC= transient loss of consciousness; OH=orthostatic hypotension. [file 12916_2023_3056_MOESM1_ESM.docx]

| **Expert committee diagnosis** | **Treating physician’s diagnosis** |
| --- | --- |
| 1 Unexplained TLOC | Unknown etiology |
| 2 Unexplained syncope | Unknown etiology |
| 2.0 Syncope due to reflex syncope or OH | Syncope due to reflex syncope or OH |
| 2.1 Reflex syncope | Syncope due to reflex syncope or OH |
| 2.1.1Vasovagal syncope | Syncope due to reflex syncope or OH |
| 2.1.2 Situational syncope | Syncope due to reflex syncope or OH |
| 2.1.3 Carotid sinus syndrome | Syncope due to reflex syncope or OH |
| 2.2 Syncope due to OH | Syncope due to reflex syncope or OH |
| 2.2.1 Initial OH | Syncope due to reflex syncope or OH |
| 2.2.2 Classic OH | Syncope due to reflex syncope or OH |
| 2.2.3 Delayed OH | Syncope due to reflex syncope or OH |
| 2.3 Cardiac syncope | Cardiac syncope |
| 2.3.1 Arrythmia (or asystole due to focal seizures) | Cardiac syncope |
| 2.3.2 Structural heart disease | Cardiac syncope |
| 2.3.3 Cardiovascular and cardiopulmonary | Cardiac syncope |
| 3 Epileptic seizure | Epileptic seizure |
| 3.1 Tonic-clonic seizure | Epileptic seizure |
| 3.2 Focal seizure* | Epileptic seizure |
| 4 Psychogenic TLOC | Psychogenic TLOC |
| 4.1 Psychogenic pseudosyncope | Psychogenic TLOC |
| 4.2 Psychogenic non-epileptic seizure | Psychogenic TLOC |
| 5 Other cause for TLOC | Other causes |
