# Supplementary figures and images for: Diagnostic and societal impact of implementing the syncope guidelines of the European Society of Cardiology (SYNERGY study)
Source: BMC Med. 2023 Sep 25;21:365. doi: 10.1186/s12916-023-03056-6 (PMC10518933; doi:10.1186/s12916-023-03056-6)

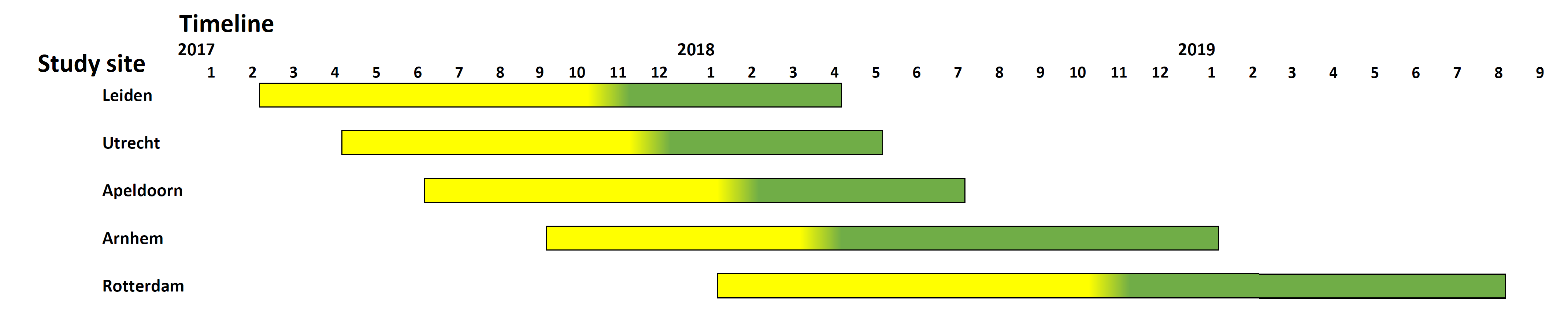

Supplement: Supplementary file 2 — Additional file 2. Study timelines. Start and end dates of the pre-intervention period (Usual Care; yellow) and the ESC Syncope Guidelines intervention (green). The figure depicts the timelines of all five study sites. After completion of the Usual Care period we paused study enrolment to educate the ED staff (yellow-green gradient). [file 12916_2023_3056_MOESM2_ESM.tif]

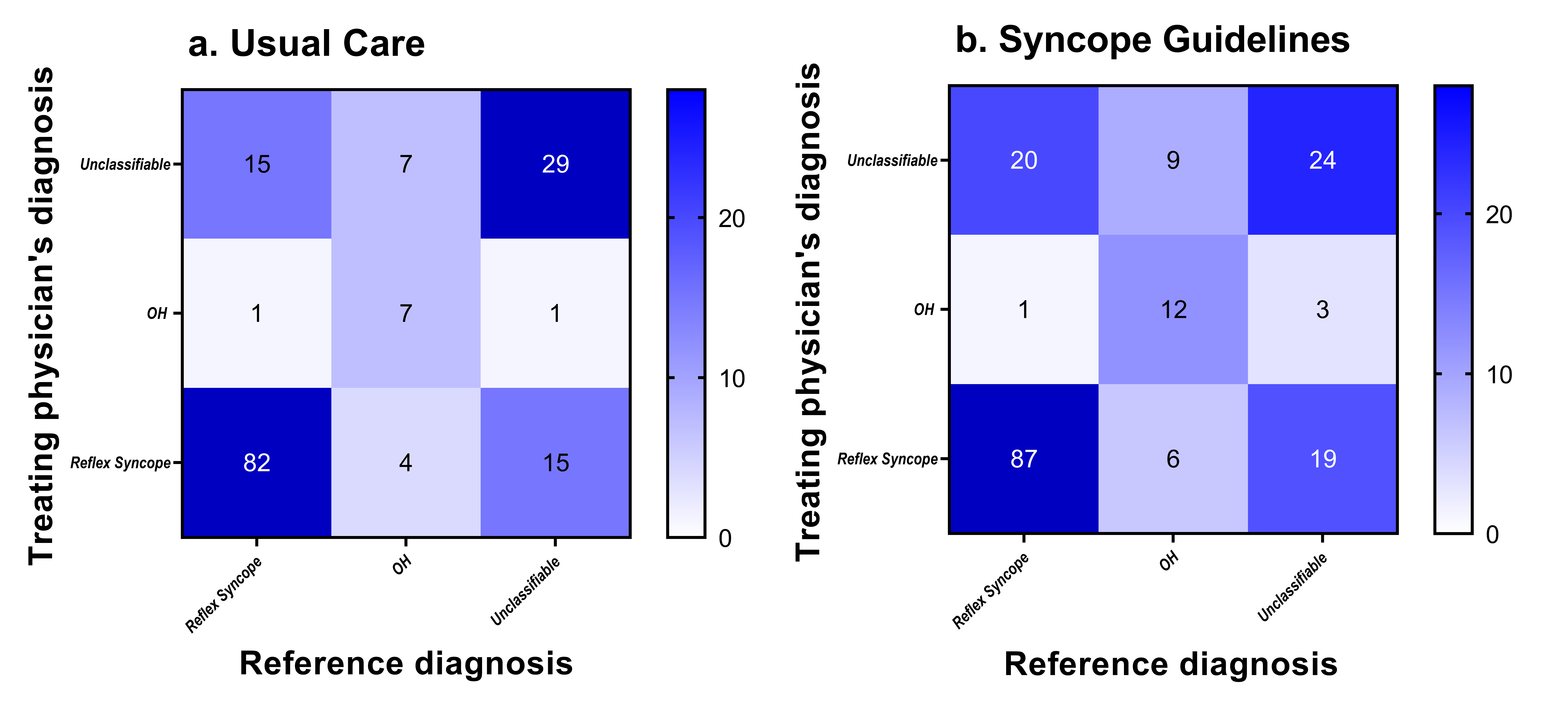

Supplement: Supplementary file 3 — Additional file 3. Heat plot expressing the concordance between the classification of blood pressure related syncope by the treating physician and the reference standard for two cohorts presenting with syncope in the emergency department: Usual Care group (left panel) and Syncope Guidelines intervention group (right panel). We selected all cases were the treating physician’s diagnosis of “blood pressure related syncope” matched the reference standard (Usual Care n=161; Syncope intervention n=181) and present the subclassifications of this category: reflex syncope, orthostatic hypotension or unclassifiable (eg, too little information to reliably differentiate or in case two conditions coincided; for instance vasovagal syncope and initial orthostatic hypotension). Abbreviations: OH = orthostatic hypotension; TLOC= transient loss of consciousness. [file 12916_2023_3056_MOESM3_ESM.tif]
